# Cancer immunotherapy in progress—an overview of the past 130 years

**DOI:** 10.1093/intimm/dxaf002

**Published:** 2025-01-10

**Authors:** Hiroaki Ikeda

**Affiliations:** Department of Oncology, Nagasaki University Graduate School of Biomedical Sciences, 1-12-4, Sakamoto, Nagasaki 852-8523, Japan; Leading Medical Research Core Unit, Nagasaki University Graduate School of Biomedical Sciences, 1-12-4, Sakamoto, Nagasaki 852-8523, Japan

**Keywords:** CAR-T therapy, immune checkpoint inhibitor, tumor heterogeneity, tumor immunology

## Abstract

Since the first approval of an immune checkpoint inhibitor, we have witnessed the clinical success of cancer immunotherapy. Adoptive T-cell therapy with chimeric antigen receptor T (CAR-T) cells has shown remarkable efficacy in hematological malignancies. Concurrently with these successes, the cancer immunoediting concept that refined the cancer immunosurveillance concept underpinned the scientific mechanism and reason for past failures, as well as recent breakthroughs in cancer immunotherapy. Now, we face the next step of issues to be solved in this field, such as tumor heterogeneity, the tumor microenvironment, the metabolism of tumors and the immune system, and personalized approaches for patients, aiming to expand the population benefitted by the therapies.

## Teaser

An overview of cancer immunotherapy

## Introduction

Here, I provide an overview of the 130-year history of the clinical development of cancer immunotherapy as well as basic science that has been trying to understand tumor immunology. I follow the long history of cancer immunotherapy and aim to reveal the reasons for the success of immune checkpoint inhibitors (ICIs) and chimeric antigen receptor T (CAR-T) cell therapy in the last 20 years. I discuss how a basic understanding of the interplay between tumors and the immune system has helped in the clinical development of cancer immunotherapy.

I also discuss the issues associated with recent cancer immunotherapy. I will try to reveal what we need to improve in future cancer immunotherapy to expand the number of patients benefitting from these endeavors.

## The hypothesis that the immune system protects the host from tumor development

**Figure 1.**
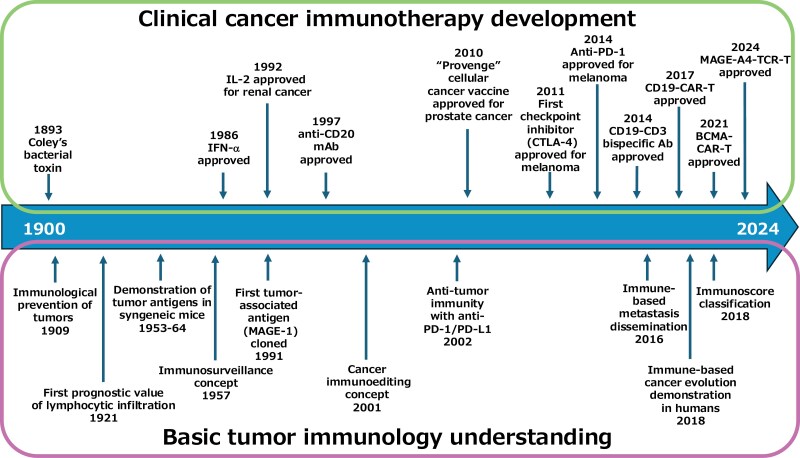
History of clinical cancer immunotherapy development and our understanding of basic tumor immunology.

Paul Ehrlich might be one of the first who described the concept that the immune system may protect the host from tumor development. He was famous as a founder of chemotherapy but also had deep knowledge in many fields, including hematology and immunology. In 1909, he envisaged that the immune system would attack not only infectious agents, but also tumor cells to protect the human body (1) (Fig. 1).

**Figure 2.**
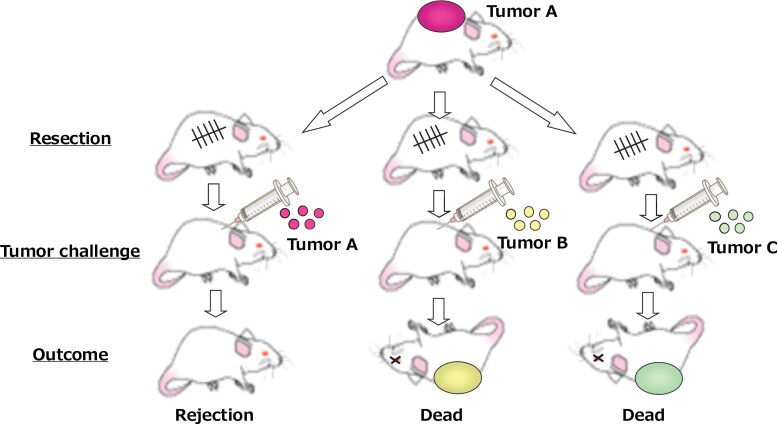
Experimental demonstration of tumor-specific antigens in syngeneic mice. A series of tumor transplantation experiments utilizing syngeneic mice revealed the existence of an immune system that recognizes and rejects the tumors as foreign agents. It was also found that every tumor has a different antigenicity, i.e. the existence of tumor-specific antigens.

From the 1950s to the 1960s, the existence of tumor antigens was experimentally demonstrated in syngeneic mice by many tumor immunologists, including Old, Foley, Prehn, Main, and Klein (2–5). The establishment of syngeneic mice made it possible for these researchers to examine whether tumor tissues could be recognized by immunity as antigenic objects, which is different from the manner in which the immune system recognizes allogeneic cells. Using syngeneic mice and chemical carcinogen-induced tumors in these mice, they found that mice treated with surgical resection of tumor A were resistant to rechallenge with tumor A. However, these mice were susceptible to challenge with independently established tumors B or C (Fig. 2). A series of experiments revealed the existence of an immune system that recognizes the tumor as a foreign agent. They also found that every tumor has a different antigenicity, i.e. the existence of tumor-specific antigens.

By these days, clinical attempts to treat cancer patients had reported the use of Coley’s bacterial toxin (in 1893) (6), hormonal therapy and radiotherapy (in 1896) (7), chemotherapy (in 1942) (8), and bone marrow transplantation (in 1957) (9, 10) (Fig. 1). Currently, the contribution of immunological mechanisms to each of these treatments is known.

Frank Macfarlane Burnet and Lewis Thomas postulated the cancer immunosurveillance concept in 1957 (11–13). They suggested that T cells might be the pivotal sentinel in the immune system’s response to cancer cells and protect the host from cancer development (Fig. 1). This concept has created debates that lasted for more than half a century. The debates on the existence of efficient immunity against tumor development lasted a long time because very limited successes on the clinical development of immunotherapy of cancer had been achieved before the 2010s.

In conjunction with the increased recognition of the importance of soluble factors such as cytokines in immune responses, interferon-α (IFN-α) was used clinically for several malignancies including chronic myeloid leukemia (14) and was approved by the Food and Drug Administration (FDA) in 1986 (15). Interleukin-2 (IL-2), which was reported as a “T-cell growth factor” in 1976 (16), was also used to treat cancer patients (17) and was approved by the FDA in 1992. However, these cytokine therapies did not improve the treatment results dramatically, and the application did not expand to various tumor types.

The first success in using a monoclonal antibody (mAb) in the treatment of patients with malignancy was achieved with the anti-CD20 mAb rituximab in patients with B-cell lymphoma (18). Rituximab was approved by the FDA for the treatment of B-cell non-Hodgkin lymphoma (B-NHL) in 1997, and later for other diseases including chronic lymphocytic leukemia (CML). Although rituximab appeared to be quite effective in these diseases, CD20 is not a tumor-specific antigen and the application of mAb therapy did not expand to other tumor types.

In 1991, Boon *et al*. cloned MAGE-1 as the first human tumor-associated antigen known to be recognized by T cells (19) (Fig. 1). Subsequently, many tumor-associated antigens have been identified. The identification of tumor-associated antigens in humans has made it possible to develop tumor-specific immunotherapies, such as cancer vaccines and cell therapy for cancer, targeting the identified antigens. However, except for the approval of Provenge in the USA as a therapeutic cancer vaccine for patients with prostate cancer in 2010 (20), a continuous failure of therapeutic cancer vaccines in clinical trials has been reported (21). The reason for the discrepancy between the significant amount of basic research results that support the existence of an immune response to cancer and difficulties in the clinical development of effective immunotherapy is largely unclear.

## The cancer immunoediting concept

One attractive clue to understanding this discrepancy came from a series of basic studies on carcinogenesis in immunodeficient mice. Schreiber *et al*. performed chemical carcinogen-induced tumor development experiments in lymphocyte-deficient RAG2 KO (recombinase-activating gene 2 knockout) mice as well as interferon-γ (IFN-γ)-insusceptible IFNGR KO (IFN-γ receptor knockout) and STAT1 KO (signal transducer and activator of transcription 1 knockout) mice (22). They observed earlier tumor formation and a higher incidence of tumor development in lymphocyte-deficient and IFN-γ-insusceptible mice compared with immunocompetent wild-type (WT) mice. They also observed an increased incidence of spontaneous tumor formation in aged RAG2 KO and RAG2 STAT1 double-KO mice.

These results showed that cancer immunosurveillance does exist. However, this was not the end of the story. They harvested tumors from carcinogen-treated WT and RAG2 KO mice groups. Tumor cells were then transplanted into WT and RAG2 KO mice. In RAG2 KO hosts, tumors derived from WT mice and RAG2 KO mice grew progressively in an equivalent manner. However, in WT hosts, 60% of the RAG2 KO mouse-derived tumors grew progressively, whereas the remaining 40% of the RAG2 KO mouse-derived tumors failed to grow and were rejected. One hundred percent of WT mouse-derived tumors grew successfully in the WT mice. These results suggested that tumors formed in the RAG2 KO host had never encountered immunological pressure; therefore, 40% of these tumor cells were eliminated when transferred into an immunocompetent environment. The immune system protects the host from highly immunogenic tumors, leaving behind less immunogenic ones.

**Figure 3.**
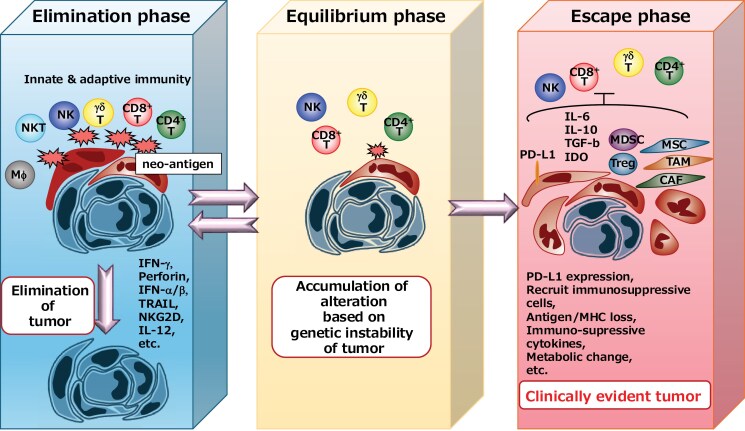
Cited from reference (65) with modifications. Cancer immunoediting concept. Many of the developing tumors were envisaged to be eliminated by the innate and adaptive immune systems in the elimination phase. Sometimes, it progresses to the equilibrium phase where tumor cells are not eliminated completely but are not able to progress. Meanwhile, genetic instability allows tumor cells to acquire additional characteristics that help tumor cells escape or suppress the immune system to enter the escape phase.

Schreiber *et al*. revealed the paradoxical effect of the immune system in selecting and facilitating the progressive growth of tumors in immunocompetent hosts. They called this process cancer immunoediting to describe the interaction between developing tumors and the immune system more precisely. They proposed three phases of cancer immunoediting: elimination, equilibrium, and escape (23) (Fig. 3). They envisaged that many of the developing tumors were eliminated by the innate and adaptive immune systems in the elimination phase. Sometimes, tumor cells are not eliminated completely but are not able to progress soon after being checked by immune cells in the equilibrium phase (24). Meanwhile, genetic instability allows tumor cells to acquire additional characteristics. When acquired alterations help tumor cells escape or suppress the immune system, they enter the escape phase, where they grow progressively without immunological pressure to form massive tumor tissue. On the basis of this concept, we can understand that many clinically evident tumors are in the escape phase, where the tumor has already acquired immunosuppressive and/or immune-escaping phenotypes. These tumors were selected to be resistant to immunological attack. In many cases, the problem is not the lack of tumor antigen, but the existence of escape mechanisms from immunological attacks. Therefore, it is difficult to develop effective cancer vaccines.

**Figure 4.**
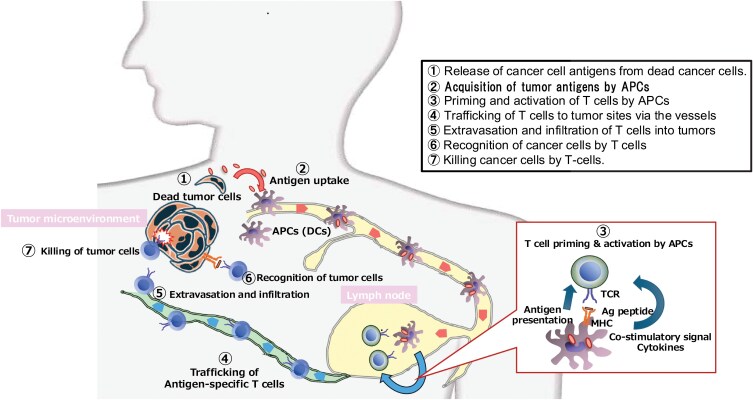
Cited from reference (65) with modifications. Cancer–immunity cycle. Chen and Mellman proposed the cancer–immunity cycle describing the process that is needed for the effective elimination of cancer by the immune system (25, 26).

Chen and Mellman proposed the cancer–immunity cycle describing the process that is needed for the effective elimination of cancer by the immune system in 2013 (25) and updated this in 2023 (26) (Fig. 4). They divide the cycle into seven steps. Step 1 is the release of cancer cell antigens from dead cancer cells. Step 2 is cancer antigen presentation to antigen-presenting cells (APCs), such as dendritic cells (DCs). Step 3 involves the priming and activation of T cells by APCs, mainly in the lymph nodes. Step 4 involves the trafficking of T cells to tumor sites via the blood. Step 5 involves extravasation and infiltration of T cells into tumors. Step 6 involves the recognition of cancer cells by T cells via the T-cell antigen receptor (TCR). Step 7 involves killing cancer cells using the cytotoxic machinery of T cells. When the cancer–immunity cycle proceeds to Step 7, it initiates a new Step 1 as a positive feedback of cancer destruction. However, they showed that there are many inhibitory factors found in every step that can stop the progression of the cancer–immunity cycle, again making us understand that we need to fight against tumors in the escape phase in a clinical setting.

## Cancer immunotherapies break through the clinical limitations

The journal *Science* selected cancer immunotherapy as “breakthrough of the year” for 2013 (27). This selection was based on the success of ICIs and adoptive T-cell therapy. Immune checkpoint molecules are receptors that deliver co-stimulatory or inhibitory signals that modulate the main signal through the TCR in T cells. Blocking the inhibitory signals from CTLA-4 (28–30) and PD-1 (31–33) showed significant anti-tumor effects in patients with various types of tumors. In 2011, an anti-CTLA-4 mAb was approved by the US FDA as the first checkpoint inhibitor for the treatment of melanoma (34, 35). In 2014, an anti-PD-1 mAb was approved for the treatment of melanoma (36) and later for the treatment of non-small cell lung carcinoma (NSCLC), renal cell carcinoma, Hodgkin’s lymphoma, and head and neck squamous carcinoma (Fig. 1). ICIs are currently used to treat many tumor types, including melanoma, NSCLC, renal cell carcinoma, Hodgkin lymphoma, head and neck carcinoma, gastric carcinoma, malignant mesothelioma, Merkel cell carcinoma, hepatocellular carcinoma, mammary carcinoma, and microsatellite instability high (MSI-H) solid tumors. T-cell exhaustion is an important obstacle in the cancer immunity. ICIs are suggested to inhibit and/or invert the exhaustion of tumor-specific T cells in the tumor microenvironment. The success of checkpoint inhibitors in the clinical treatment of cancer eloquently emphasizes the importance of overcoming the immunosuppressive and/or immune-escaping phenotype of clinical tumors in the escape phase.

Following the success of ICIs, adoptive cell therapy with T cells engineered to express a CAR gene that consists of a single-chain Fv region (scFv) of an antibody that is specific to the tumor cell surface molecule and the intracellular signal domains of CD3ζ and co-stimulatory molecules such as CD28 or 41-BB showed remarkable clinical responses. CD19-CAR-T cell (CD19-specific chimeric antigen receptor T cell) therapy was approved for the treatment of acute lymphocytic leukemia and diffuse large B-cell lymphoma in 2017 (37–40) (Fig. 1). B-cell maturation antigen (BCMA) CAR-T cells were approved by the FDA for the treatment of patients with multiple myeloma in 2021 (41–43). Blinatumomab, a CD19–CD3 bispecific antibody, exerts its anti-leukemic effect by engaging leukemia cells and host T cells in a manner that resembles a CAR-T attack. Blinatumomab was approved for the treatment of B-cell acute lymphocytic leukemia in 2014 (44). TCR-T cell therapy that utilizes T cells engineered to express TCR α and β genes derived from T-cell clones specific to tumor antigens has shown a significant tumor response in clinical trials for synovial cell sarcoma patients, targeting the NY-ESO-1 antigen (45–47). MAGE-A4-specific TCR-T cell therapy was approved by the FDA as a treatment of patients with synovial cell sarcoma in 2024. These adoptive cell therapies are effective, presumably because they can bypass several steps in the cancer–immunity cycle, where inhibitory factors may have limited the generation of a large number of effector T cells in patients. Again, this reminds us of how to fight malignancy in the escape phase.

## Issues in current cancer immunotherapy

Although ICI therapies are currently used in many kinds of cancer types including hematopoietic malignancies and solid tumors, as mentioned above, the patients who benefited from the therapies remain between 10% and 30% in most of the tumor types. The expansion of the population that can be treated by ICI is an urgent issue. Development of therapies targeting new molecules including LAG3, TIM3, and TIGIT is in progress. Combination therapy with other therapy such as cytotoxic agents and molecular targeted-based agents, or multiple ICI may expand the application.

Selection of appropriate patients is also an important issue in ICI therapy. Although biomarkers such as PD-1 ligand 1 (PD-L1) and tumor mutation burden have been used, more indicators are needed for the precise selection of patients that can be benefited. In addition, adverse events should be prevented, diagnosed early, and treated effectively with new technologies.

Although CAR-T cell therapy is starting to benefit patients with hematopoietic malignancies, especially lymphoid malignancies, the development of effective CAR-T cells against solid tumors has been largely hampered (44, 48). Table 1 highlights the problems that must be solved for the development of effective CAR-T cells against hematopoietic malignancies and solid tumors.

**Table 1.** Current issues in CAR-T cell therapy

Issue	Solid tumors	Hematopoietic malignancy
Lack of appropriate antigens	+++	++ Non-lymphoid malignancy
Tumor heterogeneity	+++ Lack of antigen/antigen presentation Cytokine sensitivity Suppressive molecule expression	+
Tumor microenvironment	+++ Suppressive cell populations (Treg, MDSC, etc.) Metabolism Vascular environment	+
Homing/migration	+++	+
T-cell fitness/function/persistence	++	++
Adverse events	++	++

A historical but ongoing issue is the lack of appropriate antigens to be targeted by CAR-T cells. Success has been achieved in targeting CD19 and BCMA; however, both are not tumor-specific but are lineage-associated antigens that are expressed in both malignant and healthy cells. Because B cells and plasma cells are dispensable, at least tentatively, the CAR-T cells against these targets effectively kill almost all tumor cells in a very efficient manner. It is difficult to identify these antigens in non-lymphoid hematopoietic malignancies. Recently, attempts to target non-lymphoid hematopoietic malignancies by CAR-T cells have been reported using CAR-T cells against the pan-leukocyte marker CD45 or myeloid markers such as FLT3, CD123, or KIT, combined with transplantation of hematopoietic stem cells that were protected from CAR-T cells by epitope base editing technology (49, 50). Identification of dispensable target antigens is also very difficult in solid tumors. Claudin 18.2-specific CAR-T cells were suggested to show tumor response in patients with gastrointestinal cancers with acceptable safety, presumably because of the polar localization of Claudin 18.2 molecule in the tight junction of the lateral side of normal epithelial cells (51).

In solid tumors, it is difficult to identify cancer-specific or cancer-associated antigens that are ubiquitously expressed in all tumor cells, reflecting the second issue of tumor heterogeneity. Tumor heterogeneity is more evident in solid tumors than in hematopoietic malignancies (52, 53). In solid tumors, tumor cells may lack or downregulate the expression of antigens and/or antigen-presenting machinery, such as β2-microglobulin.

Among other issues, a lack of sensitivity to cytokines such as IFN-γ or tumor necrosis factor-α (TNF-α) and the expression of suppressive molecules such as PD-L1 are also reported frequently. The microenvironment of solid tumors is often immunosuppressive (54, 55), as it is often infiltrated by regulatory T cells (Tregs), tumor-associated macrophages (TAMs), and myeloid-derived suppressor cells (MDSCs) (56). The metabolic environment is often severe for effector T cells (57) and preferable for cancer cells and Tregs. The vascular environment in solid tumors is not conducive to T cells. To reach a solid tumor, CAR-T cells also require homing/migration capacity to tumor sites that requires appropriate migration mechanisms and interactions between chemokines and chemokine receptors (58).

T-cell exhaustion is an important issue. As CAR-T cells and TCR-T cells can utilize non-exhausted T cells derived from patients or a third party, these modalities may have advantages compared to other therapies that rely on heavily exhausted patients’ tumor-specific T cells. Nevertheless, exhaustion of CAR-T cells has been reported as one of the obstacles in failure of the therapy. Preventing or inverting the exhaustion of CAR-T cells is an important future challenge. CAR-T cells may acquire improved fitness/function/persistence when genetically engineered or when their culture system is modulated (59).

The personalization of immunotherapy will be an important issue not only in CAR-T cell therapy but also in all cancer immunotherapies. Heterogeneity and differences always exist not only within tumors but also between individuals. Every tumor in different individuals differs in its environment, microbiota, host immune system, escape mechanism of the tumor, and style of tumor heterogeneity. How can we approach and overcome the issue of personalization? The precision medicine approach is one way to go (56, 60–62). The usefulness of the immunoscore classification has been reported (63, 64) (Fig. 1). Combination immunotherapy is an attractive method for improving the effects in some patient fractions. However, the immune system does not always assist patients. Recently, immune-based metastasis and cancer evolution have been reported (Fig. 1).

What more can we do for patients with cancer? As outlined in this article, lessons from history reveal the importance of intercommunication between basic science and clinical testing. We hope that continuous and enthusiastic communication between basic and clinical attempts will help in the development of novel and effective cancer immunotherapies that will benefit patients in the future.

## Conclusions

History tells us the importance of intercommunication between the development of clinical cancer immunotherapy and the basic understanding of tumor immunology. The next issues to be addressed in this field are tumor heterogeneity, tumor microenvironment, metabolism, and personalized approaches to patients aiming to expand the population benefitted by the therapy.

## References

[1] Ehrlich PU. ber den jetzigen stand der karzinomforschung. Ned Tijdschr Geneeskd 1909;5:273–90.

[2] Foley EJ. Antigenic properties of methylcholanthrene-induced tumors in mice of the strain of origin. Cancer Res 1953;13:835–7. 13116120

[3] Prehn RT , Main JM. Immunity to methylcholanthrene-induced sarcomas. J Natl Cancer Inst 1957;18:769–78. 13502695

[4] Klein G , Sjogren HO, Klein E, et al Demonstration of resistance against methylcholanthrene-induced sarcomas in the primary autochthonous host. Cancer Res 1960;20:1561–72. 13756652

[5] Pravtcheva DD , DeLeo AB, Ruddle FH, et al Chromosome assignment of the tumor-specific antigen of a 3-methylcholanthrene-induced mouse sarcoma. J Exp Med 1981;154:964–77. https://doi.org/10.1084/jem.154.3.964 7276830 PMC2186453

[6] Coley WB. The treatment of malignant tumors by repeated inoculations of erysipelas: with a report of ten original cases. Am J Med Sci 1893;105:487–511. 1984929

[7] Beatson GT. On the treatment of inoperable cases of carcinoma of the mamma: suggestions for a new method of treatment, with illustrative cases. Trans Med Chir Soc Edinb 1896;15:153–79. PMC5518378 29584099

[8] Fenn JE , Udelsman R. First use of intravenous chemotherapy cancer treatment: rectifying the record. J Am Coll Surg 2011;212:413–7. https://doi.org/10.1016/j.jamcollsurg.2010.10.018 21247779

[9] Thomas ED , Lochte HL Jr, Lu WC, et al Intravenous infusion of bone marrow in patients receiving radiation and chemotherapy. N Engl J Med 1957;257:491–6. https://doi.org/10.1056/NEJM195709122571102 13464965

[10] Simpson E , Dazzi F. Bone marrow transplantation 1957-2019. Front Immunol 2019;10:1246. https://doi.org/10.3389/fimmu.2019.01246 31231381 PMC6560153

[11] Burnet M. Cancer; a biological approach. I. The processes of control. Br Med J 1957;1:779–86. https://doi.org/10.1136/bmj.1.5022.779 13404306 PMC1973174

[12] Thomas L. Discussion. In: Lawrence HS (ed.), Cellular and Humoral Aspects of the Hypersensitive States. New York: Hoeber-Harper, 1959, 529–32.

[13] Burnet FM. The concept of immunological surveillance. Prog Exp Tumor Res 1970;13:1–27. https://doi.org/10.1159/000386035 4921480

[14] Talpaz M , McCredie KB, Mavligit GM, et al Leukocyte interferon-induced myeloid cytoreduction in chronic myelogenous leukemia. Blood 1983;62:689–92. 6192858

[15] Rieger PT. Interferon-alpha: a clinical update. Cancer Pract 1995;3:356–65. 15859166

[16] Morgan DA , Ruscetti FW, Gallo R. Selective in vitro growth of T lymphocytes from normal human bone marrows. Science 1976;193:1007–8. https://doi.org/10.1126/science.181845 181845

[17] Lotze MT , Frana LW, Sharrow SO, et al In vivo administration of purified human interleukin 2. I. Half-life and immunologic effects of the Jurkat cell line-derived interleukin 2. J Immunol 1985;134:157–66. 3871099

[18] Maloney DG , Liles TM, Czerwinski DK, et al Phase I clinical trial using escalating single-dose infusion of chimeric anti-CD20 monoclonal antibody (IDEC-C2B8) in patients with recurrent B-cell lymphoma. Blood 1994;84:2457–66. 7522629

[19] van der Bruggen P , Traversari C, Chomez P, et al A gene encoding an antigen recognized by cytolytic T lymphocytes on a human melanoma. Science 1991;254:1643–7. https://doi.org/10.1126/science.1840703 1840703

[20] Kantoff PW , Higano CS, Shore ND, et al; IMPACT Study Investigators. Sipuleucel-T immunotherapy for castration-resistant prostate cancer. N Engl J Med 2010;363:411–22. https://doi.org/10.1056/NEJMoa1001294 20818862

[21] Vansteenkiste JF , Cho BC, Vanakesa T, et al Efficacy of the MAGE-A3 cancer immunotherapeutic as adjuvant therapy in patients with resected MAGE-A3-positive non-small-cell lung cancer (MAGRIT): a randomised, double-blind, placebo-controlled, phase 3 trial. Lancet Oncol 2016;17:822–35. https://doi.org/10.1016/S1470-2045(16)00099-1 27132212

[22] Shankaran V , Ikeda H, Bruce AT, et al IFNg and lymphocytes prevent primary tumour development and shape tumour immunogenicity. Nature 2001;410:1107–11. https://doi.org/10.1038/35074122 11323675

[23] Dunn GP , Bruce AT, Ikeda H, et al Cancer immunoediting: from immunosurveillance to tumor escape. Nat Immunol 2002;3:991–8. https://doi.org/10.1038/ni1102-991 12407406

[24] Koebel CM , Vermi W, Swann JB, et al Adoptive immunity maintain occult cancer in a equilibrium state. Nature 2007;450:903–7. https://doi.org/10.1038/nature06309 18026089

[25] Chen DS , Mellman I. Oncology meets immunology: the cancer-immunity cycle. Immunity 2013;39:1–10. https://doi.org/10.1016/j.immuni.2013.07.012 23890059

[26] Mellman I , Chen DS, Powles T, et al The cancer-immunity cycle: Indication, genotype, and immunotype. Immunity 2023;56:2188–205. https://doi.org/10.1016/j.immuni.2023.09.011 37820582

[27] Couzin-Frankel J. Breakthrough of the year 2013. Cancer immunotherapy. Science 2013;342:1432–3. 24357284 10.1126/science.342.6165.1432

[28] Brunet J , Denizot F, Luciani MF, et al A new member of the immunoglobulin superfamily–CTLA-4. Nature 1987;328:267–70. https://doi.org/10.1038/328267a0 3496540

[29] Dariavach P , Mattei MG, Golstein P, et al Human Ig superfamily CTLA-4 gene: chromosomal localization and identity of protein sequence between murine and human CTLA-4 cytoplasmic domains. Eur J Immunol 1988;18:1901–5. https://doi.org/10.1002/eji.1830181206 3220103

[30] Leach DR , Krummel MF, Allison JP. Enhancement of anti-tumor immunity by CTLA-4 blockade. Science 1996;271:1734–6. https://doi.org/10.1126/science.271.5256.1734 8596936

[31] Ishida Y , Agata Y, Shibahara K, et al Induced expression of PD-1, a novel member of the immunoglobulin gene superfamily, upon programmed cell death. EMBO J 1992;11:3887–95. https://doi.org/10.1002/j.1460-2075.1992.tb05481.x 1396582 PMC556898

[32] Freeman GJ , Long AJ, Iwai Y, et al Engagement of the PD-1 immunoinhibitory receptor by a novel B7 family member leads to negative regulation of lymphocyte activation. J Exp Med 2000;192:1027–34. https://doi.org/10.1084/jem.192.7.1027 11015443 PMC2193311

[33] Iwai Y , Ishida M, Tanaka Y, et al Involvement of PD-L1 on tumor cells in the escape from host immune system and tumor immunotherapy by PD-L1 blockade. Proc Natl Acad Sci USA 2002;99:12293–7. https://doi.org/10.1073/pnas.192461099 12218188 PMC129438

[34] Wolchok JD , Neyns B, Linette G, et al Ipilimumab monotherapy in patients with pretreated advanced melanoma: a randomised, double-blind, multicentre, phase 2, dose-ranging study . Lancet Oncol 2010;11:155–64. https://doi.org/10.1016/S1470-2045(09)70334-1 20004617

[35] Hodi SF , O’Day SJ, McDermott DF, et al Improved survival with ipilimumab in patients with metastatic melanoma. N Engl J Med 2010;363:711–23. 20525992 10.1056/NEJMoa1003466 PMC3549297

[36] Galluzzi L , Kroemer G, Eggermont A. Novel immune checkpoint blocker approved for the treatment of advanced melanoma. Oncoimmunology 2014;3:e967147. https://doi.org/10.4161/21624011.2014.967147 25941597 PMC4292712

[37] Maude SL , Frey N, Shaw PA, et al Chimeric antigen receptor T cells for sustained remissions in leukemia. N Engl J Med 2014;371:1507–17. https://doi.org/10.1056/NEJMoa1407222 25317870 PMC4267531

[38] Schuster SJ , Bishop MR, Tam SS, et al Tisagenlecleucel in adult relapsed or refractory diffuse large B-cell lymphoma. N Engl J Med 2019;380:45–56. 30501490 10.1056/NEJMoa1804980

[39] Neelapu SS , Locke FL, Bartlett NL, et al Axicabtagene ciloleucel CAR T-cell therapy in refractory large B-cell lymphoma. N Engl J Med 2017;377:2531–44. https://doi.org/10.1056/NEJMoa1707447 29226797 PMC5882485

[40] Locke LL , Neelapu SS, Bartlett NL, et al Phase 1 results of ZUMA-1: a multicenter study of KTE-C19 anti-CD19 CAR T cell therapy in refractory aggressive lymphoma. Mol Ther 2017;25:285–95. 28129122 10.1016/j.ymthe.2016.10.020 PMC5363293

[41] Friedman KM , Garrett TE, Evans JW, et al Effective targeting of multiple B-cell maturation antigen-expressing hematological malignances by anti-B-cell maturation antigen chimeric antigen receptor T cells. Hum Gene Ther 2018;29:585–601. https://doi.org/10.1089/hum.2018.001 29641319 PMC5930946

[42] Sommer C , Boldajipour B, Kuo TC, et al Preclinical evaluation of allogeneic CAR T cells targeting BCMA for the treatment of multiple myeloma. Mol Ther 2019;27:1126–38. https://doi.org/10.1016/j.ymthe.2019.04.001 31005597 PMC6554542

[43] Young RM , Engel NW, Uslu U, et al Next-generation CAR T-cell therapies. Cancer Discov 2022;12:1625–33. https://doi.org/10.1158/2159-8290.CD-21-1683 35417527 PMC9262817

[44] Przepiorka D , Ko CW, Deisseroth A, et al FDA approval: blinatumomab. Clin Cancer Res 2015;21:4035–9. https://doi.org/10.1158/1078-0432.CCR-15-0612 26374073

[45] Ishihara M , Kitano S, Kageyama S, et al NY-ESO-1-specific redirected T cells with endogenous TCR knockdown mediate tumor response and cytokine release syndrome. J ImmunoTher Cancer 2022;10:e003811. https://doi.org/10.1136/jitc-2021-003811 35768164 PMC9244667

[46] Robbins PF , Morgan RA, Feldman SA, et al Tumor regression in patients with metastatic synovial cell sarcoma and melanoma using genetically engineered lymphocytes reactive with NY-ESO-1. J Clin Oncol 2011;29:917–24. https://doi.org/10.1200/JCO.2010.32.2537 21282551 PMC3068063

[47] Robbins PF , Kassim SH, Tran TL, et al A pilot trial using lymphocytes genetically engineered with an NY-ESO-1-reactive T-cell receptor: long-term follow-up and correlates with response. Clin Cancer Res 2015;21:1019–27. https://doi.org/10.1158/1078-0432.CCR-14-2708 25538264 PMC4361810

[48] Watanabe K , Kuramitsu S, Posey AD Jr, et al Expanding the therapeutic window for CAR T cell therapy in solid tumors: the knowns and unknowns of CAR T cell biology. Front Immunol 2018;9:248. 30416506 10.3389/fimmu.2018.02486 PMC6212550

[49] Wellhausen N , O’Connell RP, Lesch S, et al Epitope base editing CD45 in hematopoietic cells enables universal blood cancer immune therapy. Sci Transl Med 2023;15:eadi1145. https://doi.org/10.1126/scitranslmed.adi1145 37651540 PMC10682510

[50] Casirati G , Cosentino A, Mucci A, et al Epitope editing enables targeted immunotherapy of acute myeloid leukaemia. Nature 2023;621:404–14. https://doi.org/10.1038/s41586-023-06496-5 37648862 PMC10499609

[51] Qi C , Gong J, Li J, et al Claudin18.2-specific CAR T cells in gastrointestinal cancers: phase 1 trial interim results. Nat Med 2022;28:1189–98. https://doi.org/10.1038/s41591-022-01800-8 35534566 PMC9205778

[52] Rosenthal R , Cadieux EL, Salgado R, et al; TRACERx Consortium. Neoantigen-directed immune escape in lung cancer evolution. Nature 2019;567:479–85. https://doi.org/10.1038/s41586-019-1032-7 30894752 PMC6954100

[53] Patel SJ , Sanjana NE, Kishton RJ, et al Identification of essential genes for cancer immunotherapy. Nature 2017;548:537–42. https://doi.org/10.1038/nature23477 28783722 PMC5870757

[54] Ahmed N , Escalona R, Leung D, et al Tumour microenvironment and metabolic plasticity in cancer and cancer stem cells: perspectives on metabolic and immune regulatory signatures in chemoresistant ovarian cancer stem cells. Semin Cancer Biol 2018;53:265–81. https://doi.org/10.1016/j.semcancer.2018.10.002 30317036

[55] Vasan N , Baselga J, Hyman DM. A view on drug resistance in cancer. Nature 2019;575:299–309. https://doi.org/10.1038/s41586-019-1730-1 31723286 PMC8008476

[56] Kumagai S , Itahashi K, Nishikawa H. Regulatory T cell-mediated immunosuppression orchestrated by cancer: towards an immuno-genomic paradigm for precision medicine. Nat Rev Clin Oncol 2024;21:337–53. https://doi.org/10.1038/s41571-024-00870-6 38424196

[57] Nathan JA. Metabolite-driven antitumor immunity. Science 2022;377:1488–9. https://doi.org/10.1126/science.ade3697 36173838

[58] Adachi K , Kano Y, Nagai T, et al IL-7 and CCL19 expression in CAR-T cells improves immune cell infiltration and CAR-T cell survival in the tumor. Nat Biotechnol 2018;36:346–51. https://doi.org/10.1038/nbt.4086 29505028

[59] Kagoya Y , Tanaka S, Guo T, et al A novel chimeric antigen receptor containing a JAK-STAT signaling domain mediates superior antitumor effects. Nat Med 2018;24:352–9. https://doi.org/10.1038/nm.4478 29400710 PMC5839992

[60] Obradovic A. Precision immunotherapy. Science 2023;379:654–5. https://doi.org/10.1126/science.adg5585 36795815

[61] Gohil SH , Iorgulescu JB, Braun DA, et al Applying high-dimensional single-cell technologies to the analysis of cancer immunotherapy. Nat Rev Clin Oncol 2021;18:244–56. https://doi.org/10.1038/s41571-020-00449-x 33277626 PMC8415132

[62] Davis-Marcisak EF , Deshpande A, Stein-O’Brien GL, et al From bench to bedside: Single-cell analysis for cancer immunotherapy. Cancer Cell 2021;39:1062–80. https://doi.org/10.1016/j.ccell.2021.07.004 34329587 PMC8406623

[63] Willis J , Anders RA, Torigoe T, et al Multi-institutional evaluation of pathologists’ assessment compared to immunoscore. Cancers (Basel) 2023;15:4045. https://doi.org/10.3390/cancers15164045 37627073 PMC10452341

[64] El Sissy C , Kirilovsky A, Lagorce Pagès C, et al International validation of the immunoscore biopsy in patients with rectal cancer managed by a watch-and-wait strategy. J Clin Oncol 2024;42:70–80. https://doi.org/10.1200/JCO.23.00586 37788410 PMC10730081

[65] Ando Y , Tsuchiya N, Arai Y, et al Clinical Oncology Update-Essentials for Medical Oncologists. 6th edn. Japanese Society of Medical Oncology: Hiroaki Ikeda, 2021, 55–61.

